# Complex magnetism of the two-dimensional antiferromagnetic Ge_2_F: from a Néel spin-texture to a potential antiferromagnetic skyrmion

**DOI:** 10.1039/d0ra09678d

**Published:** 2021-02-24

**Authors:** Fatima Zahra Ramadan, Flaviano José dos Santos, Lalla Btissam Drissi, Samir Lounis

**Affiliations:** LPHE, Modeling & Simulations, Faculty of Science, Mohammed V University in Rabat Rabat Morocco f.ramadan88@gmail.com; Peter Grünberg Institut, Institute of Advanced Simulation, Forschungszentrum Jülich, JARA D-52428 Jülich Germany; CPM, Centre of Physics and Mathematics, Faculty of Science, Mohammed V University in Rabat Rabat Morocco; Faculty of Physics, University of Duisburg-Essen 47053 Duisburg Germany

## Abstract

Based on density functional theory combined with low-energy models, we explore the magnetic properties of a hybrid atomic-thick two-dimensional (2D) material made of germanene doped with fluorine atoms in a half-fluorinated configuration (Ge_2_F). The Fluorine atoms are highly electronegative, which induces magnetism and breaks inversion symmetry, triggering thereby a finite and strong Dzyaloshinskii–Moriya interaction (DMI). The magnetic exchange interactions are of antiferromagnetic nature among the first, second and third neighbors, which leads to magnetic frustration. The Néel state is found to be the most stable state, with magnetic moments lying in the surface plane. This results from the out-of-plane component of the DMI vector, which seems to induce an effective in-plane magnetic anisotropy. Upon application of a magnetic field, spin-spirals and antiferromagnetic skyrmions can be stabilized. We conjecture that this can be realized *via* magnetic exchange fields induced by a magnetic substrate. To complete our characterization, we computed the spin-wave excitations and the resulting spectra, which could be probed *via* electron energy loss spectroscopy, magneto-Raman spectroscopy or scanning tunneling spectroscopy.

## Abstract 2

Based on density functional theory combined with low-energy models, we explore the magnetic properties of a hybrid atomic-thick two-dimensional (2D) material made of germanene doped with fluorine atoms in a half-fluorinated configuration (Ge_2_F).

## Graphical abstract

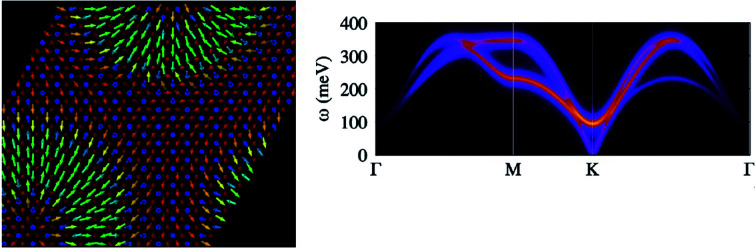

## I. Introduction

The realization of complex spin-textures hinges on the presence of competing magnetic interactions, which are heavily explored in various materials. While hosting fundamentally exciting phenomena, such magnetic states have great potential in spintronics with possible impact on information technology. For example, going beyond the conventional ferromagnetic (FM) materials for practical applications, such as the antiferromagnetic (AFM) ones^1–4^ can have various interesting advantages. AFMs are expected to be robust against perturbation due to magnetic fields, they produce no stray fields, display ultrafast dynamics, and are capable of generating large magnetotransport effects.^1^

The emergence of complex magnetic states is favored by the presence of competing magnetic interactions, which can lead to frustration. Spin–orbit driven interactions, such as the Dzyaloshinskii–Moriya interaction (DMI), also favor non-collinearity with the additional injection of a potential chiral magnetic behavior. Indeed, broken inversion symmetry and spin–orbit coupling triggers a finite DMI, which stabilizes a unique sense of rotation of the magnetic moments. Various chiral spin-swirling states can then be produced, such as chiral spin spirals, chiral domain walls or magnetic skyrmions. The latter are topological protected vortex lines in which the spins point in all the directions wrapping a sphere,^5–7^ which are promising for potential high-density and low-power spintronics technology.^8–10^ In this field, there is currently a great interest in going beyond FM skyrmions by discovering AFM skyrmions,^11–18^ which would combine the advantages of skyrmions^19–25^ and AFM properties.

The goal of this manuscript is to prospect the presence of chiral complex spin textures in two dimensional (2D) magnetic materials. They not only offer unique physical and chemical properties, but also an unprecedented flexibility in system design. When grown in a multilayer fashion, their flexibility stems from van-der-Waals bonding between neighboring atomic-thick layers of potentially very different properties, which permits virtually unlimited combinations and stackings of individual layers. The resulting properties, usually conveyed *via* proximity effects, can be very distinct from the original building block materials. Most of 2D materials do not exhibit DMIs because of their centrosymmetric crystal structure.

To break such a symmetry, some approaches consist on creating 2D structures within which different atoms are mixed in an alternating manner to generate one-atom thick hybrids.^26,27^ Other strategies such as applying a bias voltage or strain are also used.^28–30^ Chemical functionalization, impurities, boundaries and defects are other efficient ways employed in 2D sheets to tune their physical properties and induce magnetic order.^29,31–35^ In particular, chemisorption using radicals such as oxygen, hydrogen or fluorine atoms on the surface of 2D honeycomb structures leads to long-range magnetism.^36–40^ Another example consists of Sn monolayer on SiC(0001) surface, where a strong spin–orbit coupling was found on the basis of a generalized Hubbard model. This mainly contributes in the formation of a nanoskyrmion state at realistic magnetic fields and temperatures.^41^

Half-functionalization is also a powerful and widely-used tool to tailor spin and magnetic behavior in 2D materials.^42,43^ A particularly interesting adatom is fluorine since it is the most electronegative element of the periodic table. Half fluorination is an exothermic adsorption that generates stable 2D hexagonal structures.^44^ In half-fluorinated graphene, C_2_F, where F-atoms form strong covalent bonds with carbon, a threshold of the antiferromagnetic–ferromagnetic instability with strong Dzyaloshinskii–Moriya interaction was predicted^46^ with the potential presence of ferromagnetic skyrmions. The latter work challenged the *ab initio* results obtained by Rudenko *et al.*,^45^ which revealed finite AFM interactions on the triangular lattice of magnetic moments, leading to the instability of the collinear magnetic ordering due to frustration and the stabilization of a 120° Néel state. Mazurenko *et al.*^46^ proposed that the direct exchange interaction between spin orbitals, not accounted in ref. 45, leads to a ferromagnetic interaction, which is capable of compensating the antiferromagnetc indirect exchange interactions in C_2_F.

Remarkably, half-fluorination can trigger opposite magnetic behavior in hybrid 2D monolayers. While half-fluorinated BN sheet is an antiferromagnetic direct semiconductor, half-fluoro-GaN monolayer shows ferromagnetic character.^47^ In silicene–graphene (SiC), interesting magnetic properties can emerge depending on which host atom (C or Si) fluorine is attached.^48^

In this paper, we study the presence of chiral spin-textures in half fluorinated germanene using density functional theory (DFT) combined with low-energy models with spin–orbit coupling in the spirit of the methodology followed by Mazurenko *et al.*^46^ We found that the Ge_2_F is antiferromagnetic with strong DMI between the first nearest magnetic germanium neighbors. The spin dynamics simulations demonstrate the stability of the antiferromagnetic Néel state, resulting from magnetic frustration. In the latter configuration, the magnetic moments lie in the surface plane, which is induced by the out-of-plane component of the DMI vector. Extremely large magnetic fields can stabilize an antiferromagnetic skyrmion. We conjecture that this can be enabled by a proximity-effect induced by an underlying magnetic substrate. Noting that magnons in 2D structures have been probed with magneto-Raman spectroscopy^49^ and scanning tunneling microscopy,^50^ we finally explore the spin-wave excitations characterizing the obtained complex spin-textures.

## II. Computational details

The electronic and magnetic properties have been obtained using the Quantum espresso code,^51^ which is based on density functional theory (DFT). Exchange and correlation effects were taken into account using the local spin density approximation (LDA). Spin–orbit (SO) coupling was included on the basis of fully relativistic pseudopotentials. In these calculations, we set the energy cutoff to 60 Ry for the plane-wave basis. For the Brillouin-zone integration a 30 × 30 × 1 Monkhorst Pack mesh was used. To avoid the artificial interactions between layers, the thickness of the vacuum space was fixed at 20 Å.

To extract the magnetic exchange interactions required for the exploration of the magnetic properties, we built a low-energy model using an effective Hamiltonian following the work of Mazurenko *et al.*:^46^1
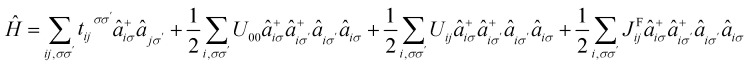
where *i*(*j*) and *σ*(*σ*′) are site and spin indices, *â*^+^_*iσ*_ (*â*_*jσ*′_) are the creation (annihilation) operators, and *U*_00_, *U*_*ij*_ and *J*^F^_*ij*_ represent local Coulomb, non-local Coulomb and non-local (*i* ≠ *j*) exchange interactions, respectively, and are obtained using the constrained random phase approximation (cRPA)^52^ as implemented in the ABINIT code.^53^*t*_*ij*_ is a hopping matrix-element taking into account the spin–orbit coupling, which is determined using the Wannier parameterization for the three nearest neighbours. To parameterize the (DFT+SO) spectra and construct the corresponding low-energy model, we use maximally localized Wannier functions, implemented in the wannier90 package.^54^

Moreover, we used the Spirit atomistic spin dynamics simulation code^55^ to solve the Landau–Lifshitz–Gilbert (LLG) equation:2
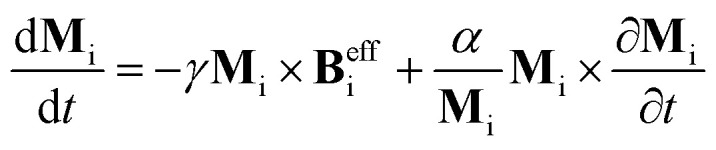
where *γ* is the electronic gyromagnetic ratio, *α* the damping factor, and **M**_i_ is the magnetic moment at a given site i. This permits the investigation of the magnetic properties of Ge_2_F described by an extended Heisenberg Hamiltonian given in eqn (5), with 
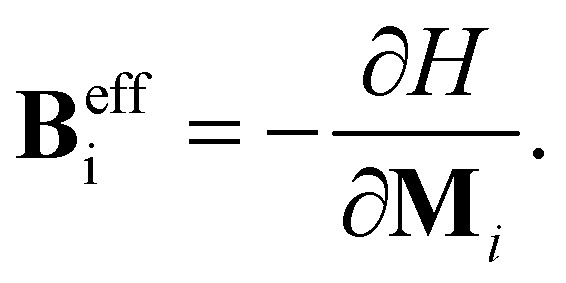
 We assume a supercell of a size of 100 × 100 × 1 atoms.

Once the ground state or a metastable state is found, we compute the adiabatic spin-wave modes and the corresponding inelastic scattering spectrum, based on time-dependent perturbation theory. The associated theoretical framework was presented in ref. 56 and ^57^ and used for various problems.^58,59^ The spin-wave eigenvalues *ω*(**k**) and eigenvectors *k* are then obtained after diagonalization of the system's dynamical matrix in the reciprocal space. We arrive to the total dynamical structure factor (summing up all the scattering channels), which is given by3
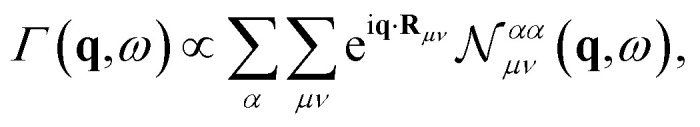
where *α*, *β* = *x*, *y*, *z* and *μ*, *ν* are site indices for spins in the unit cell that encompasses the noncollinear ground state magnetic structure. The spin–spin correlation tensor can be expressed using the information about the spin-wave modes as4
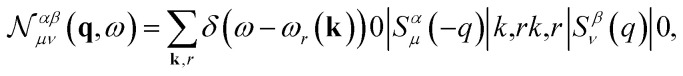
where *ω*_r_(**k**) is the energy of the spin-wave mode r with wavevector **k**, and matrix elements of the spin operators between the ground state and the excited spin-wave states.^57^

Within this framework we have access to several distinct scattering channels. In this work we present results for the total inelastic scattering spectrum due to spin waves (the sum over all scattering channels), as one would measure in an experiment with an unpolarized scattering experiment such as electron energy loss spectroscopy (EELS). The various scattering channels will also be analyzed, which could be detected *via* the recent theoretical proposal, spin-resolved EELS (SREELS), shown in ref. 57. Within the latter, a spin-polarized beam of electrons is used to probe the magnetic material. The scattered electrons are then spin-filtered with the spin analyzer collinear with the incident beam polarization. This gives rise to four scattering channels, one for each possible combinations of [incoming spin]–[outgoing spin]. Two of these channels correspond to non-spin-flip processes, namely the up–up and the down–down channels. The other two, up-down and down-up, account for spin-flip events, where angular momentum is exchanged with the sample.

## III. Results and discussion

**Fig. 1:**
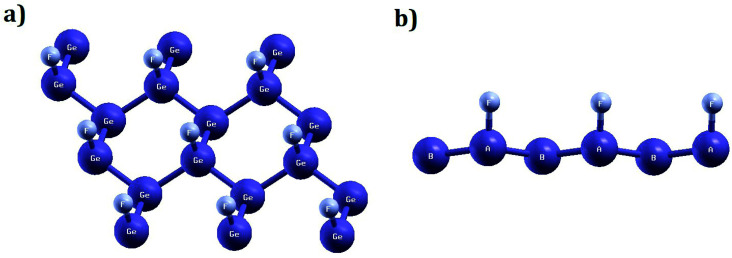
Optimized configuration (a) top (b) side of half-fluorinated germanene Ge_2_F. Fluor (small spheres) atoms binds to Germanium atoms (site A), while Germanium atoms at site B are undecorated (as shown in the side view configuration).

In this paper, we study the magnetic properties of 2D half-fluorinated germanene where F-atoms bind Ge-atoms occupying the A-sites of each hexagonal lattice while B-sites remain undecorated as shown in Fig. 1. The bond lengths are *d*_Ge–Ge_ = 2.52 Å and *d*_Ge–F_ = 1.80 Å. The structure is slightly puckered, with a buckling parameter of 0.74 Å. The interatomic angles ranging between 111.70° and 111.80° indicate an sp^3^ hybridization between Ge atoms. According to ref. 60, the Ge_2_F is an antiferromagnetic semiconductor, with a small gap energy.

**Fig. 2:**
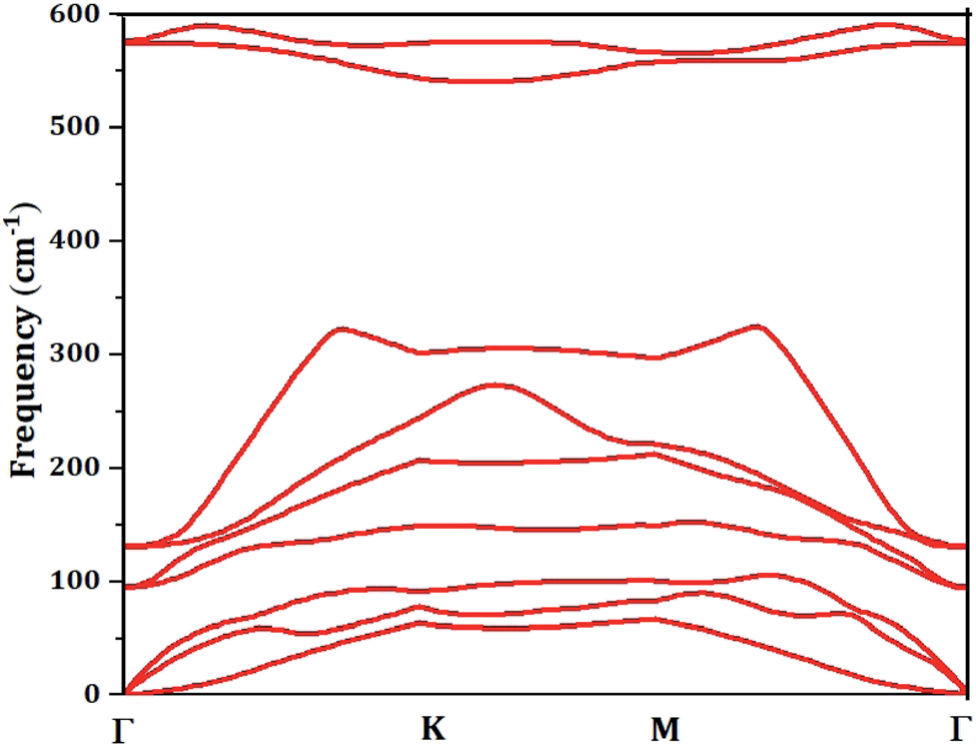
Phonon dispersion spectra calculated for the half fluorinated germanene.

To check and examine the stability of free-standing monolayer materials, various computational methods can be used such as molecular dynamics,^61,62^ the computation of formation energy and binding energy,^60^ as well as translational symmetry based on the relaxation of a finite nanocluster.^63,64^ In this work, the stability of half fluorinated germanene, confirmed in ref. 60 calculating the formation energy, is rechecked through the analysis of the phonon dispersion, the Fig. 2 displays the phonons dispersion. Analysis of the phonon spectrum shows the absence of imaginary frequency along the high-symmetry directions of the Brillouin zone for all phonon branches. It is a signature of stability of our material.

**Fig. 3:**
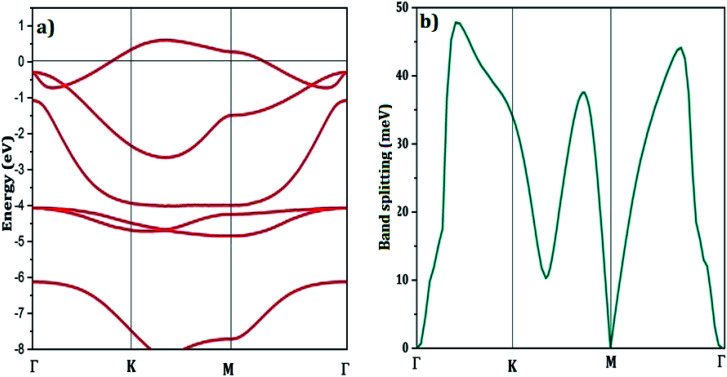
(a) The LDA band-structure with respect to the Fermi energy of half fluorinated germanene including spin–orbit coupling. Similarly to ref. 46, the impact of spin–orbit coupling on the band crossing the Fermi energy is monitored in terms of the band splitting (b).

Fig. 3a illustrates the LDA band-structure, including SO coupling. The metallic character of Ge_2_F is in good agreement with recent work.^66^ As expected, a small gap energy of 0.19 eV is reported for Ge_2_F using the generalized gradient approximation (GGA).^60^ It is worth noting that the standard DFT approximations, namely the LDA and the GGA, are known to successfully describe the ground-state properties and to underestimate the results of excited states. Thus to include quasiparticle corrections, which reproduce a band gap in accordance with the experimental measurements, one should use the GW approximation that goes beyond the scope of this work.^67,68^Fig. 3(a) also shows the bands around the Fermi level slightly overlap with other bands at the Γ-point similarly to what was found for the half fluorinated graphene (C_2_F)^46^ but with a larger spin–orbit splitting induced by the heavier Ge atoms. The splitting characterizing the band crossing the Fermi energy reaches a maximum of 48 meV, as shown in Fig. 3(b), which is larger than 38 meV, the maximum value reported for the half-fluorinated graphene C_2_F.^46^

**Fig. 4:**
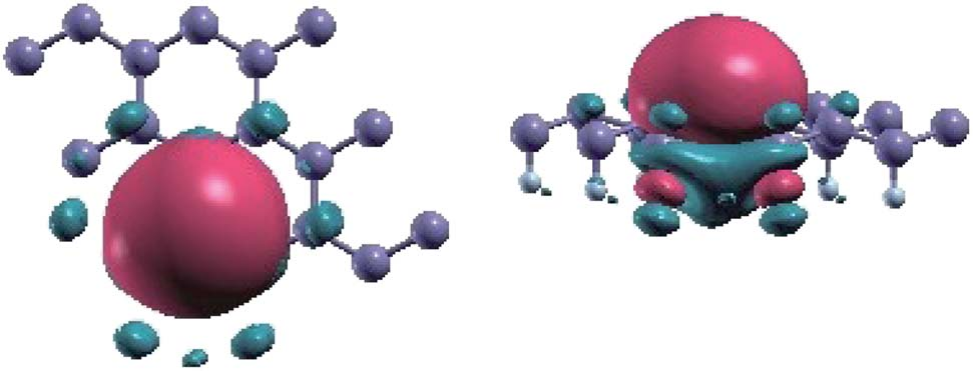
Representation of Maximally-Localized Wannier functions obtained from the projection of the *p*_*z*_ band at the Fermi level on the non-decorated Ge.

For the bands located at the Fermi level, Fig. 4 reveals that the Wannier functions, obtained from the projection of the p_*z*_ orbitals on the non-fluorinated Ge-atoms, are positioned at the centre of these atoms. The spread value of 2.04 indicates the delocalization of the Wannier function in real space. Besides, as shown in Fig. 4, the Wannier functions overlap on three nearest neighboring (NN) germanium decorated sites, giving rise to the Coulomb contribution to the total exchange interaction.

**Fig. 5:**
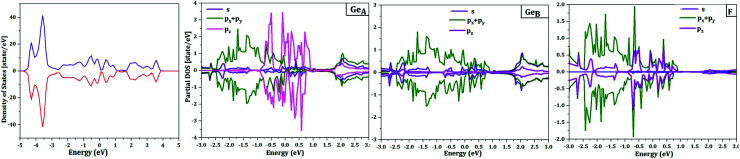
Total density of states (DOS) and partial density of states of half-fluorinated germanene as obtained with the antiferromagnetic (AFM) state described in the main text and shown in Fig. 6. The upper pannel corresponds to the majority-spin (up) channel, while the lower one hosts the minority-spin (down) channel.

The spin up/down channel of the partial density of states, displayed in Fig. 5, shows that the magnetism, which is relevant for Ge_2_F near the Fermi level, is principally originated from p_*z*_ orbitals of the non-functionalized Ge atoms. This is due to the broken π–bonding network of pure non magnetic germanene. More precisely, in Ge_2_F, the F-atoms form strong bonds with saturated Ge-atoms leaving p_*z*_ electrons of the non-decorated Ge-atoms free and localized.

**Fig. 6:**
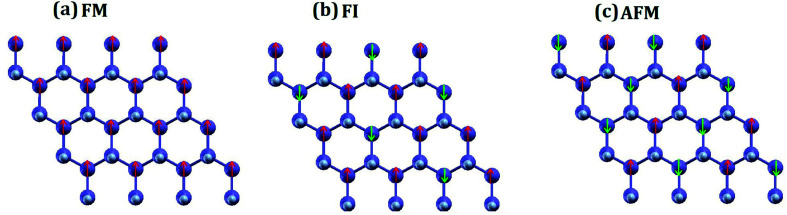
Three possible magnetic configurations of half-fluorinated germanene: (a) ferromagnetic, (b) ferrimagnetic and (c) antiferromagnetic, as explored from *ab initio*.

Within the *ab initio* formalism, we explored various magnetic configurations of Ge_2_F, namely, antiferromagnetic (AFM), ferromagnetic (FM), and ferrimagnetic (FI), utilizing the 4 × 4 supercell shown in the Fig. 6. The AFM state is found to be the lowest in energy. The energy differences with respect to the non-magnetic (NM) state are: *E*_NM_ − *E*_AFM_ = 16.93 meV, *E*_NM_ − *E*_FI_ = 15.78 meV and *E*_NM_ − *E*_FM_ = 10.85 meV.

**Fig. 7:**
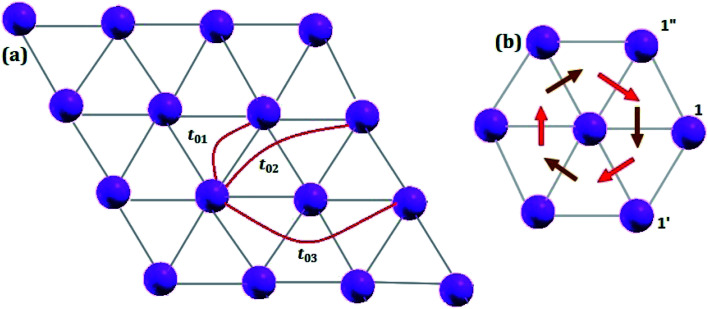
Schematic representation of (a) Hoping paths and (b) Dzyaloshinskii–Moriya vectors for half fluorinated germanene. Dark and light red arrows are DM-vectors with negative and positive z-components, respectively.

Owing to the presence of spin–orbit coupling, the hopping integrals are 2 × 2 matrices in spin-space. They are listed below in meV for the case of first NN (01), second NN (02) and third NN (03) germanium atoms (see the schematic representation in Fig. 7(a)):
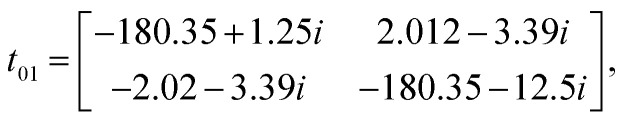

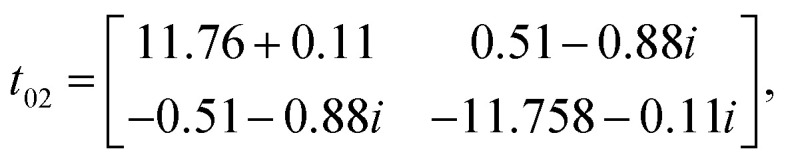

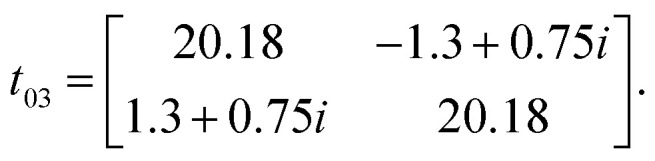


We note that (i) the hopping matrix between the first nearest neighbors contain large imaginary and non-diagonal elements, which are responsible for the antisymmetric anisotropic exchange interactions (DMI), and (ii) *t*_*ij*_ ≪ *U*. In this case, Heisenberg Hamiltonian can be constructed within the superexchange theory^69^ as follows:^70^5
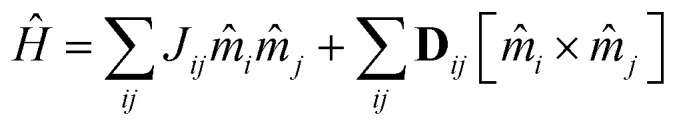
where 
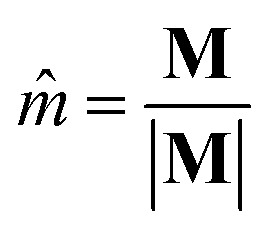
 is the classical Heisenberg vector of unit length, *J*_*ij*_ and **D**_*ij*_ are the isotropic exchange coupling and the DMI vector, respectively. The summation runs twice over all pairs.

### A Isotropic exchange interaction

A mapping of the previous Heisenberg Hamiltonian to eqn (1) leads to the following form of the isotropic exchange interaction:6
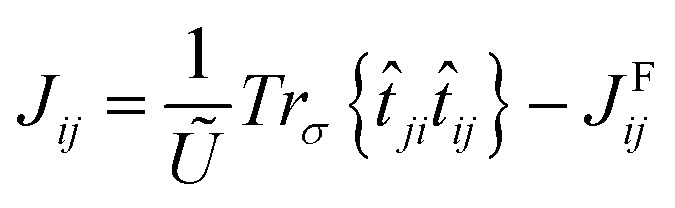
where *t̂*_*ij*_ is the hopping energy taking into account spin–orbit coupling and *Ũ* = *U*_00_ − *U*_01_ (ref. 46) corresponds to the effective local partially-screened Coulomb interaction calculated *via* constrained random phase approximation (cRPA).^52^*U*_*ij*_ and *J*^F^_*ij*_, with *i* ≠ *j*, are often much smaller than *U*_00_, which usually motivates their neglect. However, Mazurenko *et al.*^46^ has shown that *J*^F^_*ij*_ needs to be taken into account when extracting the magnetic exchange interactions in C_2_F and C_2_H. Our analysis of the case of Ge_2_F shows that in contrast to C_2_F, the non-local *J*^F^_*ij*_ are negligible. This can be explained by the extremely weak spin-polarization of the non-fluorinated Ge atoms, which play an important role in mediating the interactions between the fluorinated Ge atoms.^65^ The local Coulomb interaction *U*_00_ and non local Coulomb interaction *U*_01_ are respectively equal to 2.80 eV and 0.92 eV, which leads to *Ũ* = 1.88 eV.

The first term in eqn (6) represents the Anderson superexchange, while the non-local *J*^F^_*ij*_ is the ferromagnetic term that could originate from the direct overlap of the neighboring Wannier functions. In contrast to C_2_F,^46^ however, *J*^F^_*ij*_ is rather negligible in Ge_2_F because of the weak magnetic moment carried by germanium. Therefore, we use in practice the usual form:7
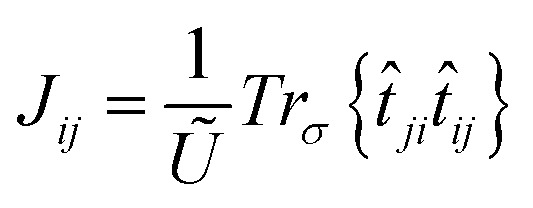


Using LDA+SO calculations by integrating the corresponding combination of the Wannier functions, one deduces that the isotropic exchange interaction between the first nearest neighbors *J*_01_ = 1.4 meV is very important when compared to the second nearest neighbors *J*_02_ = 0.11 meV and the third nearest neighbors *J*_03_ = 0.15 meV. The positive sign *J*_01_ confirms that the half fluorinated-germanene is antiferromagnetic.

### B Dzyaloshinskii–Moriya interaction

The anisotropic exchange interaction resulting from the spin–orbit interaction, is expressed as follows:8
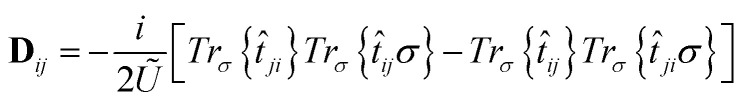
where *σ* are the Pauli matrices. For the nearest neighbour bonds in G_2_F, the DMI vectors as well as the radius vectors are presented in Table 1 and Fig. 7. In general, the orientation of DMI is defined by the symmetries of the crystal. In our case, the spin Hamiltonian symmetry is consistent with the *C*_3v_ point group of the triangular lattice formed by non-functionalized Ge atom as shown in Fig. 7b. The vertical reflections pass through the middle of bonds between the nearest neighbours. Furthermore, the corresponding DMI vectors lie in the reflection planes and are perpendicular to the interatomic bonds. The z-components of the DMI vector can change sign depending on the pair of nearest neighboring atoms.

**Table 1:** The Dzyaloshinskii–Moriya vectors

Bond	Radius vectors	*D*_*ij*_ (meV)
0–1′	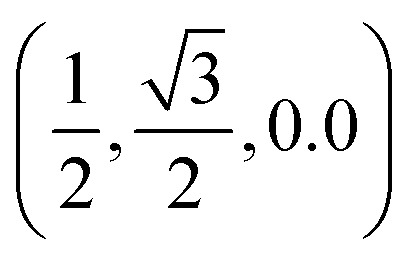	(−0.65, −0.38, 0.242)
0–1′′	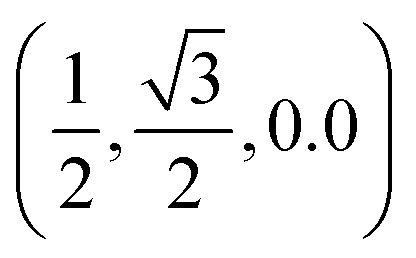	(0.65, −0.38, 0.242)
0–1	(1, 0, 0)	(0.00, −0.753, −0.242)

### C Spin-dynamics simulations

**Fig. 8:**
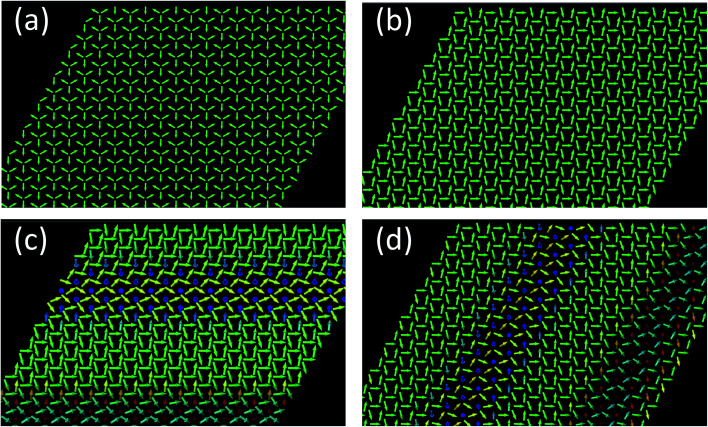
(a) The ground state of half fluorinated germanene (Ge_2_F) is a Néel antiferromagnetic state. The spins lay in the film plane due to the *z*-components of the Dzyaloshinskii–Moriya vectors (*E* = −23.21 mev per atom). (b) Applying an external field (4000 T) along the *x*-direction (to the right-hand side), a Néel distorted state is obtained (*E* = −38.63 mev per atom). (c and d) Under the influence of the same external field, metastable spin-spiral states can be obtained (*E* = −38.41 and *E* = −38.35 mev and atom, respectively).

**Fig. 9:**
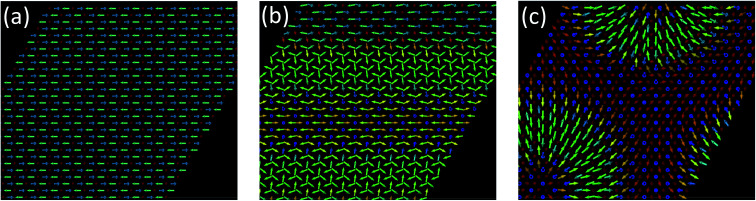
(a) Ground state of half fluorinated germanene (Ge_2_F) when the out-of-plane components of the Dzyaloshinskii–Moriya vectors are disregarded (energy of −21.97 meV per atom). The spins lay in the *x*–*z* plane. (b) Even in the absence of external, spin-spiral states can be obtained (energy of −21.942 meV per atom). (c) Applying an external field of 2000 T along the *z*-direction (to the right-hand side), an antiferromagnetic skyrmion lattice is formed (energy of −25.897 meV per atom).

After solving the LLG equation, eqn (2), utilizing the extracted magnetic exchange interactions, we obtained as the ground state a Néel state with a zero net magnetization as shown in Fig. 8(a). The nearest neighboring AFM interactions favor the realization of such a magnetic state. Interestingly, we find that the z-component of the DMI imposes to have the moments in the surface plane. If one removes the z-component of the DMI, the resulting Néel state is characterized by out-of-plane components of the magnetic moments (Fig. 9(a)). In this particular case, a metastable spin-spiral state can be stabilized (Fig. 9(b)) which has an energy of 0.031 meV per atom above that of the ground state.

When applying a large magnetic field (up to 4000 T) along the *x*-axis, applied to the right in Fig. 8b, a modified Néel state is obtained if keeping the z-component of the DMI finite. However, one can also obtain at higher energies the spin spirals shown in Fig. 8(c and d). Without the z-component of the DMI, an antiferromagnetic skyrmion can even be stabilized with a field of 2000 T along the *z*-axis (Fig. 8(c)). We conjecture that such large magnetic fields can be induced *via* a proximity effect if the 2D material is deposited on a magnetic substrate. The equivalent magnetic exchange energy for the 2000 T field is 32.41 meV, which could potentially be accessed.

**Fig. 10:**
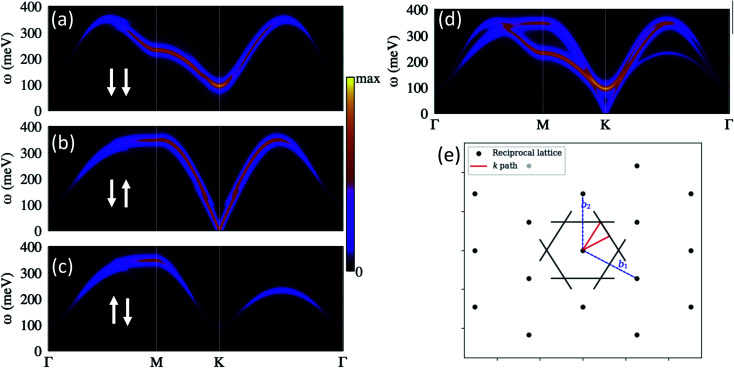
Inelastic electron scattering spectra for the Néel ground state. (a–c) Show the spin-resolved scattering channels for probing electrons polarized perpendicularly to the surface. (a) Corresponds to a non-spin-flip channel where we observe a linearly polarized mode (with zero net angular momentum). The spin-wave mode observed in (b) requires a spin-flip process. (d) Displays the total spectrum that also corresponds to a spectroscopy with unpolarized electrons. The scattering spectra was calculated along the red path indicated in (e).

The spin-excitations spectra corresponding to the Néel ground state [Fig. 8(a)] as they would be measurable with SREELS or EELS are depicted in Fig. 10(a–d). We computed the spectra along the Brillouin-zone path indicated in Fig. 10(e). Fig. 10(d) shows the total inelastic spectrum as they would be probed by an unpolarized electronic beam.

Notice the strong scattering intensities around the K point and the vanishing intensities at the Γ, which are common spectroscopy features of antiferromagnets.^58,59^ We can count up to three spin-wave branches which can be individually detected through the spin-resolved spectroscopy. Fig. 10(a–c) represent the spin-resolved spectra that arise from the possible spin orientations of the incoming and outgoing electrons. We chose the polarization of the probing electrons perpendicular to the magnetic film. Fig. 10(a) corresponds to the non-spin-flip scattering channels, such that when we send electrons with spin down we measure only scattered electrons with the same spin. The spectrum displays a single spin-wave branch with an energy minimum (100 meV) at the K point. In Fig. 10(b) and (c), we have the spectra for the excitations which require spin flips of the probing electrons. In these processes, angular momenta is exchange between the probing electrons and the spin waves. The spin-wave mode in Fig. 10(b) has a linear dispersion at low energies, which are the characteristical spin-wave feature of antiferromagnets (see for example ref. 59).

## IV. Conclusion

To summarize our study, we performed an *ab initio* investigation of the complex magnetic properties of a half-fluorinated germanene (Ge_2_F) and use a low-energy model to map the first-principles calculations and extract the magnetic exchange interactions as well as the Dzyaloshinskii–Moriya interaction vector. The latter is induced by the strong spin–orbit coupling of Germanium atoms and by the fact that the fluorine atoms break inversion symmetry.

The magnetic exchange interactions are antiferromagnetic among the first, second and third nearest neighbors, which stabilize a Néel state where the magnetic moments are lying in the surface plane. This particular configuration is favored by the out-of-plane component of the DMI vector. Antiferromagnetic spin spirals are found as metastable states once a magnetic field is applied. Interestingly, if the out-of-plane component of the DMI vector is set to zero, antiferromagnetic skyrmions can be found. For the realization of such chiral magnetic textures, we propose to use a potential magnetic substrate to induce the requested large magnetic fields. Finally, we explored for completeness the spin-waves excitations and presented the spectra that could be measurable with electron energy loss spectroscopy or its spin-resolved version.

## Conflicts of interest

There are no conflicts to declare.

## Supplementary Material
